# Use of vitamin K antagonists for secondary stroke prevention depends on the treating healthcare provider in Germany – results from the German AFNET registry

**DOI:** 10.1186/s12883-015-0371-8

**Published:** 2015-08-05

**Authors:** Karl Georg Haeusler, Andrea Gerth, Tobias Limbourg, Ulrich Tebbe, Michael Oeff, Karl Wegscheider, András Treszl, Ursula Ravens, Thomas Meinertz, Paulus Kirchhof, Günter Breithardt, Gerhard Steinbeck, Michael Nabauer, on behalf of the AFNET registry investigators

**Affiliations:** Center for Stroke Research Berlin and Department of Neurology, Charité-Universitätsmedizin Berlin, Campus Benjamin Franklin, Hindenburgdamm 30, D-12200 Berlin, Germany; Medical Hospital I, Ludwig-Maximilians-University, Munich, Germany; Institut für Herzinfarktforschung, Ludwigshafen, Germany; Department of Cardiology, Hospital Detmold, Detmold, Germany; Department of Medicine I, Brandenburg Municipal Hospital, Brandenburg, Germany; Department of Medical Biometry and Epidemiology, University Medical Center Hamburg-Eppendorf, Hamburg, Germany; Department of Pharmacology and Toxicology, Medical Faculty, Dresden University of Technology, Dresden, Germany; Department of Cardiology/Angiology, University Hospital Eppendorf, Hamburg, Germany; Department of Cardiovascular Medicine, University Hospital Münster, Münster, Germany; University of Birmingham Centre for Cardiovascular Sciences and SWBH and UHB NHS Trusts, Birmingham, UK

**Keywords:** Atrial fibrillation, Ischaemic stroke, Secondary stroke prevention, German AFNET registry, Anticoagulants

## Abstract

**Background:**

Anticoagulation using vitamin K antagonists (VKAs) significantly reduces the risk of recurrent stroke in stroke patients with atrial fibrillation (AF) and is recommended by guidelines.

**Methods:**

The German Competence NETwork on Atrial Fibrillation established a nationwide prospective registry including 9,574 AF patients, providing the opportunity to analyse AF management according to German healthcare providers.

**Results:**

On enrolment, 896 (9.4 %) patients reported a prior ischaemic stroke or transient ischaemic attack. Stroke patients were significantly older, more likely to be female, had a higher rate of cardiovascular risk factors, and more frequently received anticoagulation (almost exclusively VKA) than patients without prior stroke history. Following enrolment, 76.4 % of all stroke patients without VKA contraindications received anticoagulation, which inversely associated with age (OR 0.95 per year; 95 % CI 0.92–0.97). General practitioners/internists (OR 0.40; 95 % CI 0.21–0.77) and physicians working in regional hospitals (OR 0.47; 95 % CI 0.29–0.77) prescribed anticoagulation for secondary stroke prevention less frequently than physicians working at university hospitals (reference) and office-based cardiologists (OR 1.40; 95 % CI 0.76–2.60). The impact of the treating healthcare provider was less evident in registry patients without prior stroke.

**Conclusions:**

In the AFNET registry, anticoagulation for secondary stroke prevention was prescribed in roughly three-quarters of AF patients, a significantly higher rate than in primary prevention. We identified two factors associated with withholding oral anticoagulation in stroke survivors, namely higher age and—most prominently—treatment by a general practitioner/internist or physicians working at regional hospitals.

**Electronic supplementary material:**

The online version of this article (doi:10.1186/s12883-015-0371-8) contains supplementary material, which is available to authorized users.

## Background

Atrial fibrillation (AF) is the most frequent clinically relevant arrhythmia worldwide and affects 1–2 % of the population. As it predominantly affects the elderly, AF prevalence is expected to increase as the population ages and the number of predisposing conditions increases [1, 2]. AF is independently associated with a threefold risk of heart failure, higher all-cause mortality, and a four to fivefold higher risk of ischaemic stroke. AF-associated strokes tend to be more disabling and life-threatening than non-cardio-embolic strokes [1, 3]. Notably, stroke risk is independent of AF pattern (paroxysmal, persistent, permanent) [4] but correlates with coexisting cardiovascular risk factors, especially prior ischaemic stroke and old age [1, 5]. AF-related stroke risk can be significantly reduced by oral anticoagulation, as stated in recent guidelines [1, 6]. However, infear of both bleeding complications and multiple drug interactions, VKAs are underused in routine clinical practice specifically, underuse has been reported in elderly AF patients, those with a prior history of stroke, paroxysmal AF, minor falls, dementia, and patients treated by a general practitioner [7–12].

The publicly-funded German Competence NETwork on Atrial Fibrillation (AFNET) established a nationwide registry with 9,574 AF patients [12]. Patients were recruited by general practitioners, internists, and cardiologists who were office-based, affiliated with specialized referral centres, or part of a community or teaching hospital. Therefore, this registry provides an exclusive opportunity to analyse clinical AF management across various healthcare levels in Germany [12].

The aims of this analysis were: (I) to characterise cardiovascular risk and antithrombotic medication profiles of AF patients with prior ischaemic stroke before enrolment to the AFNET registry; (II) to determine factors associated with withholding oral anticoagulation in stroke survivors with AF, including the potential impact of the type of the treating healthcare provider.

## Methods

The design of the multicentre prospective observational registry of the German AFNET has been previously described in detail [13]. Briefly, 9,574 patients able to give written informed consent, aged ≥ 18 years and with AF documented using ECG or Holter-ECG recording—either at the time of enrolment or within the preceding 12 months—were consecutively enrolled between February 2004 and March 2006 by 191 nationwide study centres (13 tertiary care cardiology centres, 59 regional hospitals, 63 office-based cardiologists, 36 office-based internists, and 23 office-based general practitioners). All participating centres agreed to consecutive enrolment of all eligible AF patients to minimize patient selection bias. Patients were managed according to local medical practice. The study was conducted in accordance with the Helsinki Declaration and approved by the ethics committee of the Ludwig-Maximilians-University Munich, Germany (April 19, 2004). Internet-based data collection via the data capture system MARVIN was hosted by the Institute for Clinical Cardiovascular Research (Munich, Germany) as previously described [13]. Current guidelines on AF management were supplied to investigators during the initiation visit to provide guidance on state-of-the-art treatment. Patient follow-up was scheduled for up to 5 years after enrolment. Hospitalised patients (*n* = 5,068; 52.9 %) as well as outpatients (*n* = 4,506; 47.1 %) were enrolled. Information on prior stroke was available in 9,545 (99.7 %) patients. Medical stroke prevention was assessed by local investigators immediately before and after the enrolment visit.

### Statistical analysis

Data analysis was performed by the Institut für Herzinfarktforschung (IHF), Ludwigshafen, Germany and by the Department of Medical Biometry and Epidemiology, University Medical Center Hamburg, Hamburg, Germany. The results were reported as absolute numbers and percentages or mean and standard deviation. The χ^2^-test was used to test differences in proportions for dichotomous characteristics and the Mann–Whitney-Wilcoxon test was applied to detect differences in the distribution of metric variables. Multivariate statistical analysis was performed at the Department of Medical Biometry and Epidemiology, University Medical Center Eppendorf, Hamburg, Germany. In order to cope with the cluster structure of the data, hierarchal models were applied with centre identity as a random effect (two-level random intercept linear mixed models). Two-level linear mixed models relying on normal assumptions were used for continuous variables and two-level random effect binomial models were used for binary variables. As a measure of strength of the cluster effect, intra-class correlations (ICC, i.e. the proportion of variance between patients attributable to centre differences) were calculated for each model. In a second step, we introduced centre type as a fixed four-level factor to the model and determined the percent reduction of the ICC as a measure of the explanatory value of centre type and tested whether the observed centre type differences were systematic using a likelihood ratio test of the two models. Subsequently, we examined the explanatory value of the distinction of inpatient and outpatient care or of cardiologist and non-cardiologist care by repeating the analyses with the corresponding two-level factors. For the binary variable “therapeutic decision for anticoagulation at discharge”, the model was extended to incorporate clinical parameters. Two-sided p-values <0.05 were considered significant. Calculations were performed with SAS, version 9.2, or Stata, version 12.

## Results

### Baseline data of registry patients with or without ischaemic stroke

Overall, 9,545 registry patients were available for this analysis, 558 (5.8 %) of them reported an ischaemic stroke, 268 (2.8 %) a transient ischaemic attack (TIA), and 70 (0.7 %) reported both events prior to enrolment, resulting in 896 (9.4 %) registry patients with prior ischaemic stroke or TIA (“stroke patients”). Of those, 16 (1.8 %) also suffered haemorrhagic stroke prior to enrolment. Baseline characteristics of AF patients with and without prior ischaemic stroke or TIA are shown in Table 1. Stroke patients were significantly older (p < 0.0001), more often female (p < 0.05), and had a higher rate of common cardiovascular risk factors compared to AF patients without prior stroke (Table 1). Moreover, AF patients with prior stroke had permanent AF more frequently (p < 0.0001) and were less likely to have had a first episode of AF (p < 0.0001) or paroxysmal AF (p < 0.0001) upon enrolment (Table 1). The mean CHADS_2_ [Congestive heart failure, Hypertension, Age ≥75, Diabetes, Stroke (doubled)] score among stroke patients was 3.7 ± 1.0.

**Table 1:** Baseline characteristics of AFNET patients

	AF patients without prior stroke or TIA *n* = 8,649	AF patients with prior stroke or TIA n = 896	p
Age, years, mean (SD) [range]	68.1 (11.1) [18–98]	71.3 (9.6) [24–94]	<0.0001
Male, % (n)	61.3 (5,306)	57.1 (512)	<0.05
Atrial fibrillation, % (n)^a^			<0.0001
First detected	11.4 (983)	6.1 (55)	<0.001
Paroxysmal	31.7 (2,737)	26.3 (236)	<0.001
Persistent	20.1 (1,739)	21.0 (188)	0.540
Permanent	34.3 (2,967)	43.1 (386)	<0.001
Unknown	2.5 (219)	3.5 (31)	0.113
Mitral valve stenosis, % (n)	2.0 (177)	3.8 (34)	<0.001
Valvular replacement, % (n)	5.1 (440)	5.7 (51)	0.44
Heart failure, % (n)	35.4 (2,877)^a^	43.7 (370)^a^	<0.0001
Diabetes mellitus, % (n)	21.1 (1,828)	27.1 (243)	<0.0001
Arterial hypertension, % (n)	68.7 (5,938)	75.8 (679)	<0.0001
Coronary artery disease, % (n)	27.2 (2,120)^a^	39.0 (309)^a^	<0.0001
Peripheral artery disease, % (n)	6.5 (537)^a^	11.0 (93)^a^	<0.0001
Hyperlipidemia, % (n)	45.2 (3,431)^a^	55.2 (424)^a^	<0.0001
Chronic renal failure, % (n)	11.0 (907)^a^	18.7 (159)^a^	<0.0001

^a^n < 8,649 or n < 896, respectively

Baseline characteristics of AF patients with or without prior ischaemic stroke or TIA on enrolment to the AFNET registry

### Secondary stroke prevention prior to enrolment to the AFNET registry

**Fig. 1:**
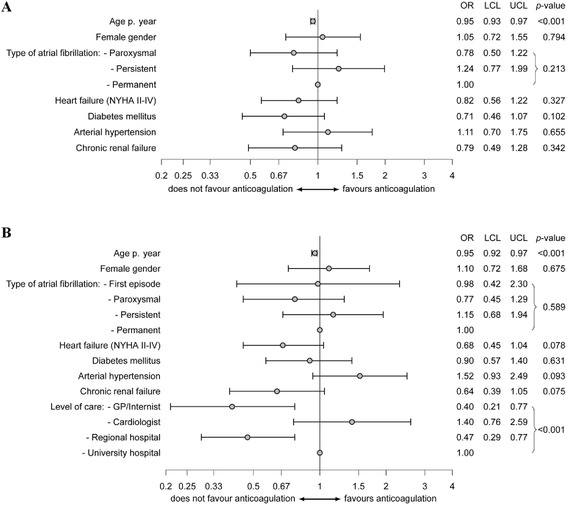
Factors associated with prescription of anticoagulants. Factors associated with prescription of anticoagulants before (**a**) and after (**b**) enrolment to the AFNET registry in AF patients with prior ischaemic stroke or TIA

AF was diagnosed prior to enrolment in 8,507 (89.1 %) of all study patients while 1,038 (10.9 %) had a first episode of AF. Excluding stroke patients with oral anticoagulation contraindications (defined as prior cerebral haemorrhage (*n* = 16), major bleeding (*n* = 37) or known malignancy (*n* = 88) and those stroke patients with a first episode of AF on enrolment (*n* = 55), and stroke patients with missing medication information (*n* = 5)), oral anticoagulation was deemed guideline-appropriate [14] in 711 (84.5 %) of 841 patients with prior stroke and known AF. Of those 711, 501 (70.5 %) AF patients received anticoagulation (VKA alone (*n* = 408), therapeutic dose heparin alone (*n* = 26), VKA plus antiplatelet drug(s) (*n* = 37), and therapeutic dose heparin plus antiplatelet drug(s) (*n* = 30)). In contrast, 125 (17.6 %) patients received antiplatelet therapy and 85 (12.0 %) no antithrombotic medication. Of those without antithrombotic medication and known CHADS_2_ score, 45 patients (61.6 %) had a CHADS_2_ ≥ 4. Compared to 2,570 stroke-free registry patients with a CHADS_2_ score of ≥ 2, in whom oral anticoagulation for primary stroke prevention was indicated according to guidelines at the time of enrolment [14, 15], stroke patients were treated significantly more frequently with anticoagulants (70.5 % vs. 61.7 %; p < 0.0001) and received no antithrombotic therapy less frequently (12.0 % vs. 17.8 %; p < 0.001). In AF patients with prior stroke, as shown in Fig. 1, only age (OR 0.95 per year; 95 % CI 0.93–0.97) was inversely associated with anticoagulant therapy before registry enrolment.

### Secondary stroke prevention at the end of the enrolment visit to the registry

Adequate information regarding medication after enrolment was available for 740 (96.7 %) of the 765 AF patients with prior stroke and without contraindications for oral anticoagulation. The majority of stroke patients (76.4 %) received anticoagulants—usually VKA (95.3 %) or heparin (therapeutic dose; 4.6 %). Overall, 10.4 % were treated with VKA or heparin and additional antiplatelets. Furthermore, 118 (15.9 %) patients were treated using antiplatelets and 57 (7.7 %) stroke patients had no medical secondary stroke prevention. Of those 57 AF patients, 31 (58.5 %) had a CHADS_2_ ≥ 4. Compared to 3,062 registry patients without prior ischaemic stroke and a CHADS_2_ score of ≥ 2, stroke patients were treated with anticoagulants more frequently (76.4 % vs. 69.2 %; p < 0.001; OR 1.44; 95 % CI 1.19–1.73) and were less likely to receive no medical stroke prevention (7.7 % vs. 10.7 %; p < 0.05; OR 0.70; 95 % CI 0.52–0.94).

According to univariate analysis (Additional file 1: Table S1), old age, a higher CHADS_2_ score, chronic renal failure, and recruitment by a physician working at a regional hospital or an office-based general practitioner/internist inversely associated with anticoagulant therapy for secondary stroke prevention after registry enrolment. After adjustment for confounders and taking the cluster structure of the data into account (Fig. 1b), only age (OR 0.95 per year; 95 % CI 0.92–0.97) and the healthcare provider remained significant factors for oral anticoagulation prescription. There were no significant differences in the rates of anticoagulation prescription between cardiologists working in university hospitals and office-based cardiologists (OR 1.40; 95 % CI 0.76–2.60), whereas office-based general practitioners/internists (OR 0.40; 95 % CI 0.21–0.77) or physicians working in regional hospitals (OR 0.47; 95 % CI 0.29–0.77) prescribed anticoagulation less frequently. As shown in Additional file 2: Table S2, the largest differences between study centres were observed across age, AF type, NYHA class, coronary artery disease, and renal dysfunction. These differences were only partially explained by centre type (Table 2, 4th column). Centre differences across AF type, NYHA class, coronary artery disease, and renal dysfunction could be sufficiently explained by differences regarding inpatients and outpatients, but this was not observed for age (Table 2, 7th column and right column). However, the heterogeneity of stroke patients treated by different healthcare providers could not be sufficiently explained by the observed differences in prescribing anticoagulation for primary or secondary stroke prevention. For patients with prior stroke, healthcare providers modestly differed in their individual anticoagulation prescriptions (intra-class correlation 12.3 %). However, guideline-concordance was primarily influenced by healthcare provider type (81.4 % explained). The observed individual healthcare provider impact was more pronounced in AF patients without prior stroke (intra-class correlation 18.2 %) whereas guideline-concordance did not seem to depend on healthcare provider type (12.4 % explained) (Table 2).

**Table 2:** Clinical parameters in 9,545 study patients with prior ischaemic stroke or TIA by type of centre

	ICC baseline	Univ. hospital vs. Regional hosp. vs. Cardiologist vs. GP/ Internist			Univ. hospital & Cardiologists vs. Reg. hosp.& GP/ Internist			Inpatients vs. Outpatients		
	[%]	Residual ICC [%]	% ex-plained	p	Residual ICC [%]	% ex-plained	p	Residual ICC [%]	% ex-plained	p
Age	25.4	19.6	23.1	<0.0001	19.8	22.2	<0.0001	25.9	0	0.18
Male	0.0002	0.00002	88.9	0.01	0.0001	55.1	0.01	0.00006	74.1	0.07
Atrial fibrillation										
First detected	12.5	6.2	50.5	0.17	10.7	14.9	0.16	7.3	41.5	0.07
Paroxysmal	9.6	7.8	18.5	0.001	9.6	0	0.19	7.3	24.0	0.02
Persistent	23.0	13.3	42.0	0.002	21.7	5.55	0.30	23.0	0	0.71
Permanent	20.2	14.4	28.7	0.08	20.3	0	0.89	15.6	22.6	0.03
CHADS_2_ score	23.0	22.2	3.7	0.047	22.0	4.33	0.01	23.4	0	0.11
Mitral valve stenosis	0.0001	0.0001	0	0.63	0.0001	9.14	0.74	0.0001	0	0.35
Valve replacement	0.0008	0.00004	94.5	0.03	0.0002	72.7	0.39	0.0001	87.4	0.01
Heart failure										
None	18.0	18.2	0	0.45	18.4	0	0.48	18.1	0	0.12
NYHA I	42.1	36.4	13.4	0.55	41.9	0.46	0.76	37.9	10.0	0.26
NYHA II	12.0	11.1	7.4	0.62	11.9	0.30	0.83	11.3	6.0	0.34
NYHA III	19.0	12.9	31.8	0.05	18.9	0.32	0.96	14.4	24.2	0.01
NYHA IV	29.0	20.0	31.2	0.003	26.9	7.4	0.14	22.3	23.0	<0.001
Diabetes mellitus	0.0006	0.00008	86.8	0.12	0.0001	77.3	0.03	0.0004	41.0	0.67
Arterial hypertension	9.6	9.5	1	0.62	9.7	0	0.31	9.9	0	0.47
Coronary artery disease	11.7	6	49.2	<0.001	9.9	15.1	0.23	7.1	39.7	0.0001
Peripheral artery disease	10.4	9.2	11.1	0.77	10.3	0.7	0.90	9.4	9.2	0.56
Chronic renal failure	4.3	3	30.7	0.005	4.7	0	0.49	2.8	34.5	<0.001
**Patients with prior stroke**										
Anticoagulation	12.3	2.3	81.4	<0.001	3.5	79.3	<0.001	12.8	0	0.08
**Patients without stroke**										
Anticoagulation	18.2	15.9	12.4	0.003	16.0	12.0	<0.001	17.9	1.41	0.07

Given are baseline intra-class correlations (ICC) as measures of the variability between centres, percent of centre variability explained by centre type or in- vs. outpatient, respectively, (“% explained”) with corresponding p value, and the residual ICC which could not be explained by the factor under consideration

## Discussion

The AFNET registry provides detailed information regarding overall AF management across a wide variety of German healthcare modalities [12, 13]. Here, we used the AFNET registry to identify factors impacting decisions regarding guideline-based anticoagulant therapy in AF patients who survived ischaemic stroke. To the best of our knowledge, comparable information is scarce, because published data from other AF registries has focused on stroke prevention in the overall cohort [12]. Moreover, this is one of the first studies to evaluate systematic, nationwide healthcare differences (including both hospital and office-based physicians) regarding stroke prevention in AF patients. Oral anticoagulation was prescribed after registry enrolment in 69.2 % of all registry patients considered eligible for anticoagulation according to the ACC/AHA/ESC 2001 guidelines [13]. This is a much higher rate than the German claims-data [16], but is similar to that of the Euro Heart Survey (69 %) and the observational Loire Valley Atrial Fibrillation Project (66 %) [17]. This may be related to the relatively high proportion of participating cardiology centres. Tertiary care cardiovascular centres and office-based cardiologists recruited approximately two-thirds of all registry patients [13] and prescribed significantly more anticoagulants than general practitioners/internists or physicians working in a regional hospital [12].

This physician-dependent under-utilization of anticoagulants in AF cohorts has been previously described for office-based general practitioners (versus cardiologists) [7, 8] but has not yet been extended to a nationwide healthcare system that includes hospital-based physicians.

The self-reported stroke rate of 9.4 % in the AFNET registry was similar to those reported in the Euro Heart Survey on Atrial Fibrillation (10.7 %), or ATRIUM [10], a prospective German AF registry that focussed on primary care. Compared to patients without a history of stroke prior to enrolment to the AFNET registry, stroke patients were significantly older, more likely to be female, and had higher rates of concomitant cardiovascular diseases (Table 1). 70.5 % of AF patients with a prior history of stroke and without contraindications for VKAs received anticoagulation before enrolment, whereas 76.4 % received VKAs following the enrolment visit. After enrolment, AF patients with a prior history of stroke received anticoagulation 1.4 times more frequently than stroke-free patients who had a CHADS_2_ score ≥ 2— i.e. indicative of anticoagulation use for guideline-adherent primary stroke prevention [14, 15]. Nevertheless, our data clearly indicate that the concomitant cardiovascular risk profile did not have a profound impact on VKA prescription in stroke patients before or after enrolment (Additional file 1: Table S1). More specifically, we found a significantly lower proportion of VKA use in older stroke patients (OR 0.95 per year; Fig. 1). Unlike the Euro Heart Survey on Atrial Fibrillation, we did not find that AF pattern played a role regarding VKA prescription rates in stroke survivors (Fig. 1) [4].

Importantly, we found that several healthcare provider modalities under-utilized anticoagulants—almost exclusively VKAs. This under-utilization of VKAs was observed for AF patients with a prior history of stroke, which is startling, as this patient subset is known to have a very high risk of recurrent stroke. If university hospital-based cardiologists were set as a reference, then office-based cardiologists (OR 1.40) prescribed significantly more guideline-concordant anticoagulation for secondary stroke prevention than office-based general practitioners/internists (OR 0.40) and regional hospitals (OR 0.47) (Fig. 1b). Though several cardiovascular risk factors varied substantially between centres and centre types (Additional file 2: Table S2)—largely explained by in- and outpatient differences—guideline-concordant anticoagulation prescription for secondary stroke prevention was mainly healthcare provider dependent. This heterogeneity could not be sufficiently explained by the observed differences between centre types. Moreover, anticoagulation prescriptions only modestly differed across individual centres within each healthcare provider group. The impact of individual healthcare providers was more pronounced in AF patients without prior stroke, whereas guideline-concordance depended less on the type of the treating healthcare provider in primary stroke prevention (Table 2). In accordance with our results, a recently published sub-analysis of the retrospective TREAT AF study—based on US health records and claims data—reported that office-based cardiologists prescribed significantly more warfarin for AF patients with a prior history of stroke (OR 1.59; 95 % CI 1.41–1.80) than primary care providers [8]. The results of the European SAFE-II study [7] are similar but the total number of stroke patients treated by office-based cardiologists was lower and an appropriate multilevel analysis including hospital-based physicians was not performed.

To both comply with the guidelines and overcome the suboptimal use of guideline-concordant oral anticoagulants in clinical practice, we recommend improved patient and physician education. The availability of today’s non-vitamin K antagonist oral anticoagulants might help achieve this goal. However, European registry data from 2012 and early 2013 indicate that VKAs are still predominantly used for AF patient anticoagulation [18, 19].

The strengths of our analysis include the large sample size of almost 10,000 registry patients with AF, the ability to discriminate between medical stroke prevention before and after enrolment to the AFNET registry, the enrolment in various centre types, as well as the consideration of the resulting cluster structure in statistical models. However, our study has several limitations. First, due to the retrospective nature of our analysis, we cannot exclude that undocumented factors have influenced physicians regarding VKA prescription, and the number of patients refusing to take VKA is unknown. Second, about 53 % of all registry patients were enrolled during an in-hospital stay. Therefore, secondary stroke prevention at hospital discharge might have been affected by diagnostic or therapeutic procedures carried out or planned in-hospital. Third, willingness to participate in the registry might inadvertently select patients and centres, as suggested by the high rate of anticoagulant use at enrolment into the AFNET registry. Therefore, we cannot comprehensively assess the implementation of antithrombotic medication for stroke prevention in AF patients in the general German population. Fourth, all registry patients were enrolled before the non-vitamin K antagonist oral anticoagulants became available.

## Conclusions

Medical secondary stroke prevention was in accordance with applicable guidelines in more than three quarters of all registry patients with prior ischaemic stroke or TIA after enrolment to the AFNET registry. Nevertheless, secondary stroke prevention was not sufficiently tailored to individual stroke risk profiles. As reported for unselected AF patients in the AFNET registry, guideline-concordant use of anticoagulants in stroke patients with AF depended on the type of the treating health-care provider, indicating an under-utilization of anticoagulants among general practitioners/internists and physicians working in regional hospitals. Counterintuitively, guideline-concordance in patients with a prior history of stroke—a group of patients at high risk of recurrent stroke -depended even more on the type of the treating health-care provider. Therefore, our data suggest that increased use of specialist care can improve stroke prevention in AF patients.

## Additional files

Additional file 1: Table S1. Guideline adherence in patients with known AF and prior ischemic stroke. Additional file 2: Table S2. Baseline characteristics according to type of enrolling centre.

## Abbreviations

GP: General practitioner
AFNET: German Competence Network on Atrial Fibrillation
AF: Atrial fibrillation
TIA: Transient ischemic attack
VKA: Vitamin K antagonist
