# Supplementary material for: Use of vitamin K antagonists for secondary stroke prevention depends on the treating healthcare provider in Germany – results from the German AFNET registry
Source: BMC Neurol. 2015 Aug 5;15:129. doi: 10.1186/s12883-015-0371-8 (PMC4524411; doi:10.1186/s12883-015-0371-8)
Supplement: Additional file 1: Table S1. — Guideline adherence in patients with known AF and prior ischemic stroke. [file 12883_2015_371_MOESM1_ESM.doc]

**Additional Table 1:**

**Guideline adherence in patients with known AF and prior ischemic stroke.** Anticoagulation in patients with known AF and prior ischemic stroke or TIA after enrolment to the AFNET registry. AF patients with contraindications to oral anticoagulation [defined as prior cerebral hemorrhage, major bleeding, or malignancy] were excluded.

| **AF patients with prior stroke/TIA** | **No anticoagulation**  n=175 | **Anticoagulation**  n=565 | **p** |
| --- | --- | --- | --- |
| Age; years; mean (SD) | 75.1 (9.6) | 69.4 (9.6) | <0.0001 |
| Male; % (n) | 50.3 (818) | 58.4 (330) | 0.06 |
| Atrial fibrillation; % (n)* |  |  | 0.83 |
| First detected | 5.7 (10) | 6.7 (38) |  |
| Paroxysmal | 25.1 (44) | 26.2 (148) |  |
| Persistent | 18.9 (33) | 22.7 (128) |  |
| Permanent | 43.4 (76) | 42.5 (240) |  |
| Unknown | 6.9 (12) | 1.9 (11) |  |
| CHADS_2_ score; mean (SD) | 4.1 (1.0) | 3.7 (1.0) | <0.0001 |
| Mitral valve stenosis; % (n) | 2.3 (4) | 4.4 (25) | 0.20 |
| Valvular replacement; % (n) | 3.4 (6) | 5.3 (30) | 0.31 |
| Heart failure; % (n) | 45.6 (73)* | 41.3 (222)* | <0.05 |
| Diabetes mellitus; % (n) | 30.9 (54) | 26.2 (148) | 0.23 |
| Arterial hypertension; % (n) | 77.7 (136) | 76.1 (430) | 0.66 |
| Coronary artery disease; % (n) | 45.8 (66)* | 36.9 (190)* | 0.05 |
| Peripheral artery disease; % (n) | 14.0 (22)* | 9.2 (50)* | 0.08 |
| Chronic renal failure; % (n) | 27.8 (45)* | 15.6 (84)* | <0.001 |
| Type of health care provider; % (n) |  |  | <0.005 |
| GP/Internist | 12.6 (22) | 6.9 (39) |  |
| Cardiologist | 13.7 (24) | 26.5 (150) |  |
| Regional hospital | 36.6 (64) | 18.1 (102) |  |
| University hospital | 37.1 (65) | 48.5 (274) |  |

* n < 175 or n < 565, respectively
