# Supplementary material for: Use of vitamin K antagonists for secondary stroke prevention depends on the treating healthcare provider in Germany – results from the German AFNET registry
Source: BMC Neurol. 2015 Aug 5;15:129. doi: 10.1186/s12883-015-0371-8 (PMC4524411; doi:10.1186/s12883-015-0371-8)
Supplement: Additional file 2: Table S2. — Baseline characteristics according to type of enrolling centre. [file 12883_2015_371_MOESM2_ESM.docx]

|  | **Overall** | **Univers.**  **Hospital** | **Regional Hospital** | **Cardiologist** | **General practitioner/ internist** | **p** |
| --- | --- | --- | --- | --- | --- | --- |
| Age; years; mean (SD) | 70.8 (9.8) | 68.8 (10.0) | 74.4 (8.8) | 70.3 (9.6) | 73.4 (8.3) | <0.0001 |
| Male; % (n) | 56.5 (418) | 62.0 (210) | 53.0 (88) | 54.6 (95) | 41.0 (25) | 0.01 |
| Atrial fibrillation; % (n) |  |  |  |  |  | <0.0001 |
| First detected | 6.7 (48) | 7.0 (23) | 11.0 (17) | 2.9 (5) | 4.9 (3) |  |
| Paroxysmal | 26.8 (192) | 32.5 (107) | 7.1 (42) | 22.1 (38) | 8.2 (5) |  |
| Persistent | 22.6 (161) | 25.5 (84) | 20.0 (31) | 11.6 (20) | 42.6 (26) |  |
| Permanent | 44.1 (316) | 35.0 (115) | 41.9 (65) | 63.4 (109) | 44.3 (27) |  |
| CHADS_2_; mean (SD) | 3.8 (1.0) | 3.7 (1.0) | 4.0 (1.0) | 3.6 (1.0) | 3.9 (1.1) | 0.02 |
| Mitral valve sten.; % (n) | 3.9 (29) | 4.1 (14) | 1.8 (3) | 4.6 (8) | 6.6 (4) | 0.34 |
| Valvul. replacement; % (n) | 4.9 (36) | 4.4 (15) | 1.2 (2) | 8.1 (14) | 8.2 (5) | 0.02 |
| Heart failure; % (n) | 42.3 (295) | 45.7 (145) | 40.0 (60) | 38.4 (64) | 44.3 (26) | 0.0001 |
| Diabetes mellitus; % (n) | 27.3 (202) | 25.4 (86) | 32.5 (54) | 23.0 (40) | 36.1 (22) | 0.07 |
| Art. hypertension; % (n) | 76.5 (566) | 74.6 (253) | 80.7 (134) | 76.4 (133) | 75.4 (46) | 0.50 |
| Coronary artery dis.; % (n) | 38.9 (256) | 47.7 (147) | 37.1 (49) | 29.5 (48) | 21.4 (44) | <0.0001 |
| Periph. artery dis.; % (n) | 10.3 (72) | 9.9 (32) | 8.8 (13) | 10.6 (18) | 15.0 (9) | 0.60 |
| Chronic renal fail.; % (n) | 18.4 (129) | 22.0 (72) | 21.6 (35) | 9.3 (14) | 13.1 (8) | <0.005 |

**Additional Table 2:**

**Baseline characteristics according to type of enrolling centre.** Baseline characteristics of AF patients with prior ischemic stroke or TIA before enrolment to the AFNET registry split by the type of enrolling centre. Nominal parameters are given as frequency (%); continuous parameters as mean ± SD. P-values indicate whether there are differences between groups.
